# Microfracture Versus Arthroscopic Debridement for the Treatment of Symptomatic Cartilage Lesions of the Knee: 2-Year Results From a Multicenter Double-Blinded Randomized Controlled Trial

**DOI:** 10.1177/03635465251346961

**Published:** 2025-06-26

**Authors:** Per-Henrik Randsborg, Tommy Frøseth Aae, Håvard Visnes, Thomas Birkenes, Jūratė Šaltytė Benth, Øystein Bjerkestand Lian, Heidi Andreassen Hanvold, Asbjørn Årøen

**Affiliations:** † Department of Orthopaedic Surgery, Akershus University Hospital, Lørenskog, Norway; ‡ Institute of Clinical Medicine, Faculty of Medicine, University of Oslo, Oslo, Norway; § Department of Orthopaedic Surgery, Kristiansund Hospital, Møre & Romsdal Hospital Trust, Kristiansund, Norway; ‖ Clinical Research Unit, Møre & Romsdal Hospital Trust, Ålesund, Norway; ¶ Department of Orthopaedics, Sørlandet Hospital Kristiansand, Kristiansand, Norway; # Oslo Sports Trauma Research Center, Norwegian School of Sport Sciences, Oslo, Norway; ** Department of Orthopaedic Surgery, Haukeland University Hospital, Bergen, Norway; †† Sports Traumatology and Arthroscopy Research Group, University of Bergen, Bergen, Norway; ‡‡ Health Services Research Unit, Akershus University Hospital, Lørenskog, Norway; §§ Department of Physiotherapy, Akershus University Hospital, Lørenskog, Norway; Investigation performed at Akershus University Hospital, Lørenskog, Norway

**Keywords:** microfracture, cartilage lesions, arthroscopic debridement, randomized controlled trial

## Abstract

**Background:**

Knee cartilage injuries can lead to significant functional limitations, pain, and diminished quality of life. Microfracture (MF) is the most common surgical procedure for smaller (<2 cm^2^) cartilage lesions of the knee. However, there is no established gold-standard surgical intervention.

**Purpose:**

To compare functional and patient-reported outcomes after MF and arthroscopic debridement (AD) for symptomatic, isolated femoral cartilage injuries <2 cm^2^ in patients aged 18 to 50 years.

**Study Design:**

Randomized controlled trial; Level of evidence, 1.

**Methods:**

A total of 65 patients were included, randomized to undergo either MF (n = 31) or AD (n = 34), and followed for 2 years. The primary outcome was the change in the Knee injury and Osteoarthritis Outcome Score (KOOS) Quality of Life subscore. Secondary outcomes included scores for the other KOOS subscales, Tegner activity scale, Lysholm score, and visual analog scale for pain.

**Results:**

The mean age at the time of inclusion was 33.2 ± 9.7 years. There were 44 (68%) male patients. The mean size of the lesion was 1.2 ± 0.6 cm^2^. There was no statistically significant difference between the groups in the change in the KOOS Quality of Life subscore from baseline to 2 years (3.5 [95% CI, –10.0 to 16.9]; *P* = .61). There were 10 complications in 5 patients in the MF group and 2 complications in 2 patients in the AD group. According to a linear mixed model, there were no statistically significant differences between the groups for any of the secondary outcomes at any time point during the 2-year follow-up period.

**Conclusion:**

MF was not superior to AD when treating femoral cartilage lesions of the knee <2 cm^2^.

Knee cartilage injuries in young active adults can lead to significant functional limitations, pain, and diminished quality of life.^19,39^ At present, there is no established gold-standard surgical intervention, resulting in a variety of proposed treatment options.^9,17,20^ Among the current methods are arthroscopic debridement (AD), microfracture (MF), mosaicplasty, autologous chondrocyte implantation (ACI), and osteochondral allografts.^9,17,20,25,35,45^

The purpose of AD is to stabilize the lesion and to remove loose intra-articular fragments and inflammatory mediators down to the subchondral bone, but not through it, and does not include any cartilage-specific involvement.^36^ MF is a surgical technique used to treat cartilage lesions in the knee, particularly in younger patients, popularized in the 1980s by Steadman et al.^46^ The technique involves creating tiny holes (“microfractures”) in the underlying bone to stimulate the formation of new cartilage. The microfractures allow bone marrow to seep into the defect, creating a superclot and bringing growth factors and stem cells into the lesion.^31^ This method aims to promote the healing process by encouraging the production of a fibrocartilage layer over the damaged area.

MF is the most common surgical procedure for cartilage lesions of the knee.^8,13,22^ Previous studies have indicated that the procedure is most effective for smaller lesions (<2 cm^2^).^28,30^ Most randomized controlled trials (RCTs) evaluating the effect of MF have compared it with other cartilage procedures.^16,18,37,49^ Despite extensive publications, the method has not been compared with a true control group, and the efficacy of the technique has therefore been questioned.^15^

The developers of MF designed a specific and detailed rehabilitation program, which according to them provides the ideal physical environment for mesenchymal stem cells to mature into a satisfactory cartilage surface and is therefore crucial for successful outcomes.^21^ However, the other cartilage procedures to which MF has been compared are also closely associated with strict and supervised rehabilitation programs.^4,29^ This raises the question of whether rehabilitation programs after knee cartilage surgery can explain the improvement observed in most published studies. To determine whether MF influences clinical and patient-reported outcomes after knee cartilage surgery beyond the effect provided by the rehabilitation protocol, we designed a double-blinded RCT. We hypothesized that there would be no statistically or clinically meaningful differences in functional and patient-reported outcomes at 2 years between patients aged 18 and 50 years treated with MF or AD with identical postoperative rehabilitation protocols for a symptomatic, isolated femoral cartilage injury <2 cm^2^.

## Methods

This study was part of the Norwegian Cartilage Project, a national multicenter collaboration between 4 university hospitals. Patients referred to any of the 4 collaborating institutions with an isolated, symptomatic full-thickness chondral defect <2 cm^2^ of the femoral condyles or trochlea were assessed for inclusion.^1^ The lesion had to be grade 3 or 4 according to the International Cartilage Regeneration & Joint Preservation Society (ICRS) classification^3,5^ with a Lysholm score <75.^7^ The size and ICRS grade of the lesion were assessed on magnetic resonance imaging and verified arthroscopically before randomization. The inclusion criteria consisted of patients aged 18 to 50 years who had a stable knee, full range of motion, normal alignment (within 5° varus or valgus measured on imaging of the hip-knee-ankle angle), no previous cartilage surgery to the knee, a body mass index <30 kg/m^2^, and no radiological signs of osteoarthritis as defined by the Kellgren-Lawrence classification.^23^ Weightbearing, fixed-flexion posteroanterior radiographs were obtained using a Synaflexer positioning frame (Synarc).^38^

The study was approved by the Regional Committee for Medical Research Ethics South East Norway (No. 2015/2022) and the Data Protection Officer at Akershus University Hospital and registered at ClinicalTrials.gov (NCT02637505). All patients provided written informed consent before inclusion.

### Randomization

The treatment allocation (MF or AD) was determined using block randomization facilitated by a computer generator (randomization.org). The allocation groups were printed on paper in faint text and placed in opaque envelopes marked with consecutive study numbers, with one set for each institution. An assistant not involved in the study handled the printing and concealment to ensure blinding.

### Surgical Procedure

All patients underwent a diagnostic arthroscopic examination to confirm the diagnosis and verify the size and ICRS grade of the lesion. Overall, 3 standard incisions were made: superolateral, anteromedial, and anterolateral. Loose bodies were removed, and any meniscal issues were addressed. The inflamed synovium was debrided. The focal cartilage lesion was measured using a standard 4-mm arthroscopic probe and graded according to the ICRS classification.^5^ Intra-articular local anesthetics were not utilized because of their potential harmful effects on cartilage.^6,26,40^

Subsequently, the randomization envelope was opened, and those assigned to the MF group underwent the procedure at the end. All surgeons were experienced in both AD and MF before treating the patients in the study and were trained in the study protocol.

#### Arthroscopic Debridement

After the diagnostic arthroscopic examination, the lesion was stabilized by debriding the edges down to the subchondral bone, without penetrating it, using a ring curette.

#### Microfracture

**Figure 1.**
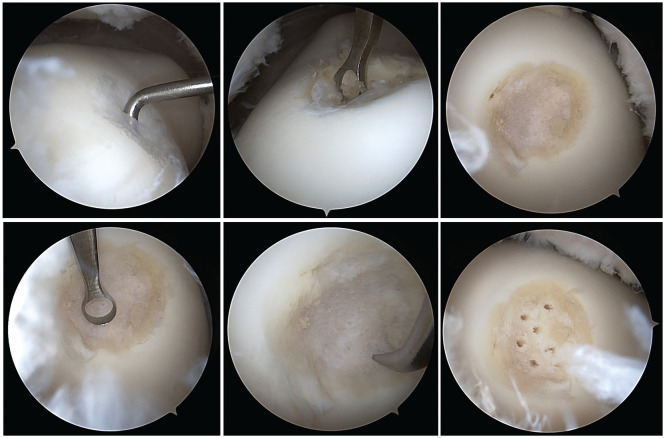
Microfracture of a 1.2-cm^2^ ICRS (International Cartilage Regeneration & Joint Preservation Society) grade 3 lesion of the trochlea in a 35-year-old male patient. Top left: Probing the lesion. Top middle: Debridement and removal of loose tissue. Top right: Stabilizing the edges. Bottom left: Removing the calcified layer with a curette. Bottom middle and right: Creating microfractures with an arthroscopic owl.

MF was performed after AD at the end of surgery in patients randomized to the MF group. The calcified cartilage layer was removed with a curette, followed by the use of a specialized angled awl to create multiple microfractures from the periphery toward the center (Figure 1). The microfractures were spaced 3 to 4 mm apart and penetrated 2 to 4 mm deep into the subchondral bone. A sufficient depth was confirmed by observing marrow fat droplets emerging from the microfractures when the fluid pump pressure was reduced. These surgical steps adhered to the protocols established by Mithoefer et al^32^ and Steadman et al.^47^

### Rehabilitation

All patients were admitted to the hospital for one night, during which a continuous passive motion machine facilitated early knee movements. After discharge, patients in both treatment groups followed the same rehabilitation program outlined by Wondrasch et al.^50^ This program emphasized active rehabilitation and education, consisting of 3 phases: accommodation, rehabilitation, and return to activity (Table 1). Patients met with a physical therapist on the day after surgery to receive instructions on range of motion exercises and restrictions for phase 1. All patients were nonweightbearing for a minimum of 2 weeks. Within 2 weeks after surgery, they were referred to a local physical therapist, who guided them through phases 1 to 3. Active rehabilitation included cardiovascular training, progressive resistance exercises for the knee and hip, and neuromuscular training, incorporating balance and plyometric exercises. The local physical therapists received guidance from the study’s physical therapist through regular communication.

**Table 1:** Rehabilitation Protocol for Both Groups

Phase	Physical Therapy and Activities	Objectives	Criteria for Progression to Next Phase
(1) Accommodation	● Education/coaching ● Ice, elevation, and compression ● Isometric exercises Range of motion ● Gait training (no weightbearing for 2 wk)	● Reduce pain and swelling ● Normalize range of motion ● Regain quadriceps control	● No pain or swelling during activities of daily living ● Flexion at 90° ● Normalized quadriceps activity while walking (clinical evaluation by physical therapist)
(2) Rehabilitation	● Stationary cycling ● Progressive knee and hip resistance training ● Neuromuscular training	● Recover full range of motion ● Normalize muscle strength ● Attain dynamic joint stability during activities of daily living	● Full range of motion ● No pain or swelling during and after training sessions ● Equally distributed weight on lower limbs during weightbearing exercises with no shift of trunk (visually assessed by physical therapist) ● Ability to stand on one limb on flat surface for at least 10 s
(3) Return to activity	● Knee and hip resistance training ● Neuromuscular training ● Cardiovascular training	● Recover strength and neuromuscular control ● Return to activity/sport	● Return to sport based on individual assessment

### Primary Endpoint

The primary endpoint was the difference in the Knee injury and Osteoarthritis Outcome Score (KOOS) Quality of Life (QoL) subscore between the groups at 2-year follow-up. The selection of the primary endpoint was based on previous studies that identified poor knee-related quality of life as a major concern for patients with cartilage issues, making it the most crucial variable for assessing treatment success.^19,39^ The KOOS is a validated patient-reported outcome measure used in cartilage research studies.^12,41^ It evaluates 5 domains: pain, symptoms, activities of daily living, sports and recreational function, and knee-related quality of life. Each domain is scored on a scale from 0 to 100, with 100 representing the best possible outcome.

### Secondary Endpoints

The secondary endpoints included a combination of validated patient-reported outcome measures, self-explanatory questionnaires, clinical parameters, and complications:

- KOOS: all subscales (except the primary endpoint: QoL subscale).
- Tegner activity scale: This is a widely used outcome measure to assess a patient’s activity level, particularly in relation to knee function.^48^ It was originally developed to evaluate the functional level of patients after knee injuries or surgery, especially for sports-related activities.
- Lysholm score: a condition-specific outcome measure widely used in orthopaedic and sports medicine research, containing 8 domains: limping, locking, pain, stair climbing, use of support, instability, swelling, and squatting.^27^
- Visual analog scale (VAS): a scale for pain in which 0 represents no pain and 10 represents the worst pain imaginable.
- Complications: Major complications were defined as any reoperation of the knee, pulmonary embolism, and sepsis or deep (intra-articular) infections. Minor complications included any unscheduled return visits to the outpatient clinic or general physician or transient pain and stiffness.

All outcome questionnaires were completed by the patients before surgery (baseline) and at each scheduled follow-up visit at 3, 6, 12, and 24 months. Complications were recorded prospectively during the 2-year follow-up period.

### Statistical Analysis

A change in the KOOS QoL subscore of 8 to 10 points has been shown to be clinically meaningful.^12,42^ The power analysis is therefore based on detecting a difference of 10 points and assuming a standard deviation for the change in the KOOS QoL subscore from baseline to 2 years of 18 points.^43^ With a power of 80% and significance level of 5%, a total of 52 patients in each group were required to demonstrate a statistically significant difference by an independent-samples *t* test.

Demographic and clinical characteristics are presented as means with standard deviations, medians with ranges (minimum-maximum), or frequencies with percentages, as appropriate. The difference between groups for the primary endpoint (change in KOOS QoL subscore from baseline to 2-year follow-up) was assessed using an independent-samples *t* test. Given the multiple measurement time points, repeated observations were available for each patient, potentially introducing within-patient correlations. To assess differences between the groups in the trends of primary and secondary endpoints, a linear mixed model with random intercepts was used. The model included fixed effects for time, group, and the interaction between time and group. Post hoc analyses, to be interpreted as exploratory, were conducted to compare the groups at each individual time point. The results are presented as means and standard deviations for each group at each time point, along with the mean differences, 95% confidence intervals (CIs), and *P* values derived from the linear mixed model. A linear mixed model employs all available data, including dropouts, and provides unbiased estimates under the assumption that data are missing completely at random, which we adopted. Complications between the groups were compared using the chi-square test. All statistical tests were 2-sided, and results with *P* values < .05 were considered statistically significant. Data analysis was performed using Stata (Version 18; StataCorp).

## Results

**Figure 2.**
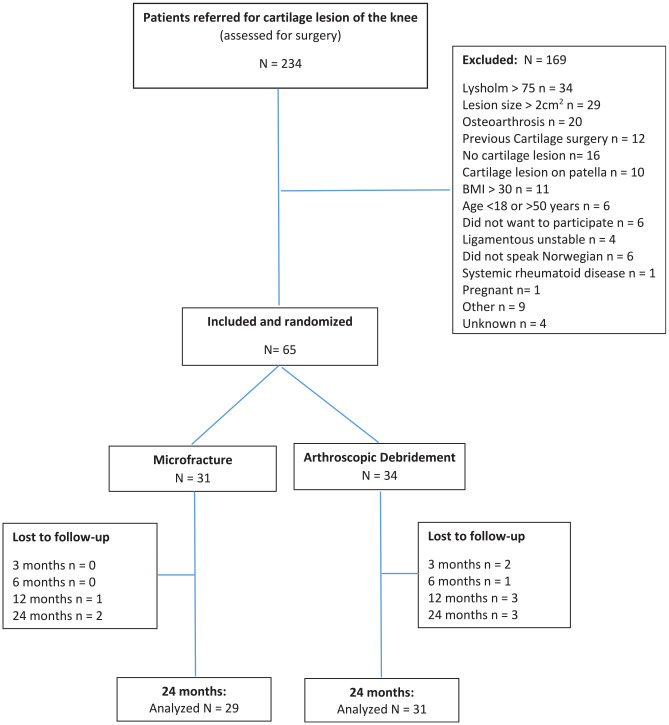
Flowchart of included patients. Overall, 3 patients missed follow-up visits in the microfracture group, while 5 different patients in the arthroscopic debridement group missed a total of 9 follow-up appointments.

From January 2016 to October 2022, there were 234 patients referred to the collaborating institutions with a symptomatic cartilage lesion of the knee, of whom 65 patients were included and randomized (Figure 2). The included patients had a mean age of 33.2 years (range, 20-49 years), with 44 (68%) of them being men. The mean lesion size was 1.2 cm^2^. A detailed summary of the patients’ characteristics at the time of inclusion is provided in Table 2.

**Table 2:** Patient Characteristics*^a^*

	Total (n = 65)	MF (n = 31)	AD (n = 34)
Age, y	33.2 ± 9.7	31.1 ± 10.0	35.1 ± 9.2
Male sex	44 (67.7)	21 (67.7)	23 (67.7)
Body mass index, kg/m^2^	25.9 ± 3.4	26.6 ± 3.6	25.4 ± 3.3
Lesion size, cm^2^	1.2 ± 0.6	1.1 ± 0.5	1.3 ± 0.6
Right knee	37 (56.9)	17 (54.8)	20 (58.8)
Operative time, min	29.8 ± 9.8	32.8 ± 10.9	27.0 ± 7.9
Anatomic location			
Lateral femoral condyle	17 (26.2)	8 (25.8)	9 (26.5)
MFC	33 (50.8)	17 (54.8)	16 (47.1)
MFC/trochlea	1 (1.5)	00 (0.0)	1 (2.9)
Trochlea	13 (20.0)	6 (19.4)	7 (20.6)
Trochlea/MFC*^b^*	1 (1.5)	00 (0.0)	1 (2.9)
Smoking			
Never	49 (75.4)	22 (71.0)	27 (79.4)
Current	5 (7.7)	2 (6.5)	3 (8.8)
Previous	11 (16.9)	7 (22.6)	4 (11.8)
Education			
Less than high school	5 (7.7)	2 (6.5)	3 (8.8)
High school	36 (55.4)	19 (61.3)	17 (50.0)
College	12 (18.5)	4 (12.9)	8 (23.5)
University (>4 y)	12 (18.5)	6 (19.4)	6 (17.7)
Symptom duration, median (range), mo	17 (2-260)	14 (2-260)	21 (2-120)

a Data are presented as mean ± SD or n (%) unless otherwise indicated. AD, arthroscopic debridement; MF, microfracture; MFC, medial femoral condyle.

b When the majority of the lesion was in the trochlea, and only a smaller part in the medial femoral condyle, we classified it as Trochlea/MFC, rather than the other way around.

### Patient-Reported Outcomes

**Figure 3.**
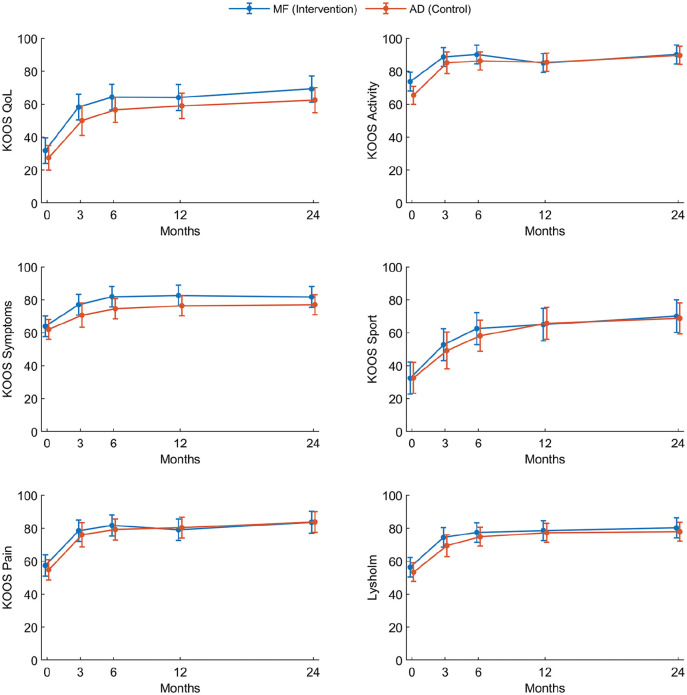
Comparison of the Knee injury and Osteoarthritis Outcome Score (KOOS) and Lysholm score between the groups during the 2-year follow-up period. The dots and I bars indicate the means and 95% confidence intervals, respectively, estimated by the linear mixed model. There were no statistically significant differences between the groups at any time points. AD, arthroscopic debridement; MF, microfracture; QoL, Quality of Life.

The improvement from baseline to final follow-up in the KOOS QoL subscore was 37.9 ± 30.2 for the MF group and 34.5 ± 21.6 for the AD group, which was statistically significant and clinically meaningful for both groups. However, the difference in improvement between the groups (3.5 [95% CI, –10.0 to 16.9] points in favor of the MF group) was neither statistically significant (*P* = .61) nor clinically meaningful (Table 3). The linear mixed model did not reveal any statistically significant differences in any outcomes at any time points during the 2-year follow-up period (Table 4 and Figure 3).

**Table 3:** Change in KOOS QoL Subscore From Baseline to 2 y*^a^*

	n	Mean ± SD	Mean Difference (95% CI)	*P* Value*^b^*
MF	29	–37.9 ± 30.2	3.5 (–10.0 to 16.9)	.61
AD	31	–34.5 ± 21.6		

a AD, arthroscopic debridement; KOOS, Knee injury and Osteoarthritis Outcome Score; MF, microfracture; QoL, Quality of Life.

b Independent-samples *t* test.

**Table 4:** Patient-Reported Outcomes*^a^*

	MF		AD		Mean Difference (95% CI)	*P* Value
	n	Mean ± SD	n	Mean ± SD		
KOOS Quality of Life						
Preoperative	31	31.9 ± 13.8	33	27.1 ± 13.0	4.4 (–6.4 to 15.3)	.42
3 mo	31	58.3 ± 23.8	31	50.8 ± 19.4	8.2 (–2.7 to 19.1)	.14
6 mo	31	64.3 ± 25.3	32	56.8 ± 20.1	7.7 (–3.1 to 18.6)	.16
12 mo	30	63.8 ± 29.7	30	59.1 ± 23.3	5.1 (–6.0 to 16.1)	.37
24 mo	29	68.8 ± 29.5	32	62.9 ± 22.2	6.7 (–4.2 to 17.7)	.23
KOOS Symptoms						
Preoperative	31	63.9 ± 15.9	34	62.1 ± 18.0	1.9 (–6.8 to 10.5)	.68
3 mo	31	77.1 ± 19.2	32	71.7 ± 16.2	6.4 (–2.4 to 15.1)	.15
6 mo	31	81.9 ± 21.1	33	75.2 ± 17.0	7.3 (–1.4 to 16.1)	.10
12 mo	30	82.4 ± 18.9	31	76.8 ± 18.8	6.2 (–2.6 to 15.0)	.17
24 mo	29	81.5 ± 19.5	32	77.2 ± 15.9	4.7 (–4.2 to 13.5)	.30
KOOS Pain						
Preoperative	31	57.4 ± 17.8	33	54.5 ± 16.7	2.7 (–6.3 to 11.7)	.56
3 mo	31	78.5 ± 21.3	31	76.8 ± 16.9	2.4 (–6.6 to 11.5)	.60
6 mo	31	81.7 ± 21.6	31	79.9 ± 16.2	2.5 (–6.6 to 11.5)	.59
12 mo	30	79.0 ± 22.5	30	80.5 ± 17.7	–1.3 (–10.4 to 7.9)	.79
24 mo	29	83.1 ± 20.9	32	84.3 ± 14.4	–0.1 (–9.2 to 8.9)	.98
KOOS Activities of Daily Living						
Preoperative	31	73.7 ± 18.7	33	65.3 ± 17.9	8.3 (0.4 to 16.1)	**.04**
3 mo	31	88.7 ± 15.0	31	85.7 ± 16.4	3.5 (–4.5 to 11.4)	.39
6 mo	31	90.2 ± 14.8	32	86.5 ± 15.7	3.9 (–4.1 to 11.8)	.34
12 mo	30	86.9 ± 18.0	30	88.1 ± 17.3	–0.5 (–8.5 to 7.5)	.91
24 mo	29	89.8 ± 15.2	32	90.4 ± 14.2	0.5 (–7.5 to 8.5)	.90
KOOS Sports and Recreation						
Preoperative	31	32.4 ± 24.1	33	32.6 ± 23.4	–0.2 (–13.7 to 13.3)	.98
3 mo	31	52.7 ± 31.9	31	50.2 ± 27.0	3.5 (–10.1 to 17.2)	.62
6 mo	31	62.6 ± 34.2	32	58.8 ± 25.0	4.4 (–9.2 to 18.0)	.52
12 mo	30	64.3 ± 33.5	30	65.7 ± 26.1	–0.8 (–14.5 to 13.0)	.91
24 mo	29	69.5 ± 31.0	32	69.4 ± 24.3	1.3 (–12.4 to 15.0)	.85
VAS knee pain while standing						
Preoperative	31	4.0 ± 2.4	34	4.2 ± 2.5	–0.2 (–1.2 to 0.8)	.73
3 mo	30	2.1 ± 2.2	32	1.9 ± 1.7	0.2 (–0.8 to 1.2)	.71
6 mo	31	2.1 ± 2.1	33	1.7 ± 1.7	0.3 (–0.7 to 1.3)	.54
12 mo	30	2.2 ± 2.5	31	1.7 ± 2.1	0.3 (–0.7 to 1.3)	.54
24 mo	29	1.5 ± 1.9	32	1.0 ± 1.4	0.3 (–0.7 to 1.3)	.53
VAS knee pain while sitting						
Preoperative	31	2.4 ± 1.9	34	2.6 ± 2.4	–0.2 (–1.1 to 0.6)	.55
3 mo	30	1.0 ± 1.2	32	1.3 ± 1.9	–0.4 (–1.2 to 0.4)	.34
6 mo	31	0.9 ± 0.9	33	1.1 ± 1.5	–0.3 (–1.1 to 0.6)	.54
12 mo	30	1.1 ± 1.3	31	1.4 ± 2.2	–0.4 (–1.3 to 0.4)	.30
24 mo	29	0.9 ± 1.3	32	1.0 ± 1.5	–0.2 (–1.0 to 0.7)	.70
Tegner activity scale						
Preoperative	31	3.0 ± 1.8	34	2.1 ± 1.7	0.9 (–0.1 to 1.8)	.09
3 mo	31	2.9 ± 2.1	32	2.5 ± 1.7	0.5 (–0.5 to 1.5)	.34
6 mo	31	3.5 ± 2.3	33	4.3 ± 2.3	–0.9 (–1.8 to 0.1)	.09
12 mo	30	3.9 ± 1.9	31	3.9 ± 2.3	–0.03 (–1.0 to 1.0)	.96
24 mo	29	4.1 ± 2.2	32	4.5 ± 2.1	–0.3 (–1.4 to 0.7)	.79
Lysholm score						
Preoperative	31	56.4 ± 11.1	34	53.3 ± 14.2	3.1 (–5.1 to 11.2)	.46
3 mo	31	74.5 ± 19.6	32	70.4 ± 17.2	5.0 (–3.2 to 13.2)	.23
6 mo	31	77.5 ± 20.1	33	75.4 ± 17.6	2.6 (–5.6 to 10.9)	.53
12 mo	30	78.4 ± 18.2	31	77.8 ± 17.0	1.3 (–7.0 to 9.6)	.76
24 mo	29	79.8 ± 19.6	32	78.1 ± 14.4	2.3 (–6.0 to 10.6)	.59

a AD, arthroscopic debridement; KOOS, Knee injury and Osteoarthritis Outcome Score; MF, microfracture; VAS, visual analog scale.

### Complications

There were 10 complications in 5 patients in the MF group and 2 complications in 2 patients in the AD group (Table 5). There was 1 deep infection overall; this was in a patient in the MF group. She subsequently underwent 3 arthroscopic washouts of the knee and long-term antibiotic treatment. After successful treatment of the infection, she underwent closed manual manipulation of the knee under anesthesia because of stiffness. Another patient in the MF group underwent arthroscopic surgery and washout because of a suspected infection, which was later classified as sterile hemarthrosis. Additionally, 2 other patients in the MF group underwent the arthroscopic removal of loose bodies. In the AD group, there was 1 reoperation for the removal of loose bodies in the knee (Table 5). The number of patients with complications was too low to perform any meaningful statistical analysis.

**Table 5:** Complications*^a^*

	MF (n = 31)	AD (n = 34)	*P* Value*^b^*
Patients with complications	5	2	**.18**
Major complications	7	1	
Reoperation (arthroscopic surgery)	5	1	
Pulmonary embolism	0	0	
Manipulation under anesthesia (arthrofibrosis)	1	0	
Deep infection	1	0	
Minor complications	3	1	
Hemarthrosis	1	0	
Unplanned follow-up within 4 wk after surgery	2	1	
Total complications	10	2	

a AD, arthroscopic debridement; MF, microfracture.

b Chi-square test.

## Discussion

Our study could not identify any observed benefit of the MF technique compared with simple AD consisting of stabilization of the lesion and the removal of synovitis and debris. Both groups improved both clinically and statistically significantly over the 2-year study period, but no statistically significant differences in clinical or patient-reported outcomes between the groups were found at any follow-up time points.

The effectiveness of MF for knee cartilage lesions has been the subject of numerous systematic reviews and meta-analyses. Mithoefer et al^30^ conducted a systematic review of both controlled and noncontrolled studies to evaluate the clinical efficacy of MF for cartilage lesions of the knee. They found that all of the included studies reported improved knee function in the first 24 months after MF and that the improvement was largest for younger patients with smaller lesions. However, 21 of the 28 included studies in the review were case series without a control group, and as such, the study could not determine whether MF is better than other treatment approaches.

The improvement seen in case series may very well be attributed to regression toward the mean. Patients typically seek treatment when their outcomes, such as pain or loss of function, are at their worst. Because of the natural variation of symptoms, clinical studies are often overrepresented by patients with unusually high pain scores. When these scores are retested during follow-up, the scores naturally regress toward the mean, regardless of treatment.^11^

Although the documentation for the effectiveness of MF is largely based on case series, MF is still the most common procedure for smaller cartilage lesions of the knee. The few RCTs that have been conducted invariably compare MF with other cartilage restoration techniques, such as ACI, matrix-induced ACI, or osteochondral allograft transplantation.^16,17,37,49^ MF has not been found to be superior to any of these techniques. In fact, when the data from these RCTs are pooled in meta-analyses, MF is found to be less effective than the other methods.^2,51^ Furthermore, because MF has never been compared with a true control group (either sham surgery or nonoperative controls), we do not know the true effect of MF on clinical and patient-reported outcomes. Nevertheless, MF has emerged as the gold standard in new cartilage procedures, potentially exerting significant influence on the perceived success or failure of new technologies. We compared MF with simple AD in which the lesion was prepared as for the MF technique but without penetrating the subchondral bone. The study was double blinded (both the patient and the assessors were blinded to the procedure). Because we did not find any differences between the groups, we conclude that recruiting “mesenchymal stem cells” by microfracturing the subchondral bone plate to create bleeding did not have any measurable additional effect for the patient beyond AD and the strict rehabilitation program. Furthermore, there were more severe complications in the MF group, including 5 reoperations and 1 deep infection, compared with only 1 reoperation for the removal of loose bodies in the AD group. Although the number of complications was too low to allow any meaningful statistical analysis, the differences in complications in our study indicate that the intra-articular bleeding caused by the MF technique may in fact be harmful. In 2016, Erggelet and Vavken^15^ proclaimed that there is limited evidence that MF should be accepted as the gold standard for the treatment of cartilage lesions of the knee joint. Some authors suggest that the limited penetration of the MF awl into the subchondral bone may reduce its effectiveness.^24,52^ This has led to the development of the nanofracture technique, which uses a smaller diameter motorized drill for deeper bone perforations.^52^ However, there is a lack of basic science and clinical studies on the effects of nanofracture drilling.

Although several trials have compared different surgical treatment options for knee cartilage lesions, none has demonstrated statistically significant differences.^33^ This suggests that the observed improvements may be attributable to postoperative rehabilitation rather than to the surgical procedure itself.^10,21,50^ All clinical trials evaluating outcomes after cartilage surgery of the knee have placed patients on a strict, intensive, and prolonged postoperative rehabilitation program. This will likely improve compliance to the rehabilitation protocol and may partly explain the clinical improvements observed. We believe that the individualized rehabilitation program in our study, supervised in close collaboration with the physical therapist, ensured good compliance and may explain why both groups improved equally. Previous studies have indicated that active rehabilitation can delay or avoid the need for cartilage surgery in patients similar to ours. Wondrasch et al^50^ implemented an active rehabilitation program in 48 patients scheduled for surgery of a focal cartilage lesion of the knee. After 3 months, there was a statistically significant and clinically meaningful improvement in the KOOS score, load progression, and hop score to the point at which 31 (65%) of the patients declined surgery for their cartilage lesion. This indicates that good short-term results can be achieved with physical therapy alone. The debridement and stabilization of the cartilage lesion that both groups received in our study may have contributed to the improvements observed. However, to determine whether arthroscopic surgery is beneficial for small cartilage lesions, further randomized trials are needed to compare AD with nonoperative management or sham surgery.

The results indicate that MF did not provide additional benefits compared with simple AD. Larger multicenter RCTs with a nonoperative control group are required to further clarify the indication for arthroscopic surgery for smaller isolated cartilage lesions.

### Limitations

The main limitation of our study was its limited power. The initial sample size calculation required 52 patients per group to detect a 10-point difference in the KOOS QoL subscore. However, newer studies suggest that a change of 15.6 points is more appropriate,^34^ which would have reduced the required sample size to 22 per group. Our study included 31 patients in the MF group and 34 patients in the AD group. No trends favoring one group were observed, and any potential differences are unlikely to have been clinically relevant, even with the intended sample size.

The study had to be stopped because of slow inclusion and the lack of funding. The strict inclusion criteria left us with few eligible patients. This is a well-known issue in cartilage research.^14,44^ The main reasons for exclusion were previous cartilage surgery or too high a body mass index.

The study was conducted in a single country, and the majority of patients were included from a single institution, limiting the external validity of the results. Furthermore, the patients were followed for only 2 years. There could be between-group differences over time: for example, in the development of osteoarthritis. However, despite these limitations, we believe that the findings are of interest to the orthopaedic community.

## Conclusion

The study demonstrated that MF did not offer additional benefits in terms of functional or patient-reported outcomes compared with simple AD for full-thickness cartilage lesions of the knee <2 cm^2^. MF was not superior to AD when treating femoral cartilage lesions of the knee.
